# Binding of CIB1 to the α_IIb_ tail of α_IIb_β_3_ is required for FAK recruitment and activation in platelets

**DOI:** 10.1371/journal.pone.0176602

**Published:** 2017-05-24

**Authors:** Meghna U. Naik, Tejal U. Naik, Ross Summer, Ulhas P. Naik

**Affiliations:** Cardeza Center for Vascular Biology, Department of Medicine, Thomas Jefferson University, Philadelphia, PA, United States of America; University of Kentucky, UNITED STATES

## Abstract

**Background:**

It is believed that activation of c-Src bound to the integrin β_3_ subunit initiates outside-in signaling. The involvement of α_IIb_ in outside-in signaling is poorly understood.

**Objectives:**

We have previously shown that CIB1 specifically interacts with the cytoplasmic domain of α_IIb_ and is required for α_IIb_β_3_ outside-in signaling. Here we evaluated the role of CIB1 in regulating outside-in signaling in the absence of inside-out signaling.

**Methods:**

We used α_IIb_ cytoplasmic domain peptide and CIB1-function blocking antibody to inhibit interaction of CIB1 with α_IIb_ subunit as well as *Cib1*^*-/-*^ platelets to evaluate the consequence of CIB1 interaction with α_IIb_ on outside-in signaling.

**Results:**

Fibrinogen binding to α_IIb_β_3_ results in calcium-dependent interaction of CIB1 with α_IIb_, which is not required for filopodia formation. Dynamic rearrangement of cytoskeleton results in CIB1-dependent recruitment of FAK to the α_IIb_ complex and its activation. Disruption of the association of CIB1 and α_IIb_ by incorporation of α_IIb_ peptide or anti-CIB1 inhibited both FAK association and activation. Furthermore, FAK recruitment to the integrin complex was required for c-Src activation. Inhibition of c-Src had no effect on CIB1 accumulation with the integrin at the filopodia, suggesting that c-Src activity is not required for the formation of CIB1-α_IIb_-FAK complex.

**Conclusion:**

Our results suggest that interaction of CIB1 with α_IIb_ is one of the early events occurring during outside-in signaling. Furthermore, CIB1 recruits FAK to the α_IIb_β_3_ complex at the filopodia where FAK is activated, which in turn activates c-Src, resulting in propagation of outside-in signaling leading to platelet spreading.

## Introduction

At sites of blood vessel injury, platelets adhere to subendothelial adhesive proteins and are activated. Upon activation, platelets change shape, secrete the intracellular granule contents, and promote fibrinogen (Fg) binding to its receptor, α_IIb_β_3_ through inside-out signaling [1]. Fg-binding induces a signaling cascade, termed outside-in signaling, that involves clustering of α_IIb_β_3_ and further reorganization of cytoskeleton, as well as the association of several signaling molecules responsible for clot retraction, which is necessary for thrombus stability [1–6].

Protein tyrosine kinases such as Src family kinases, Syk, and FAK are activated during outside-in signaling [7, 8]. Src have been shown to be constitutively associated with the β_3_ tail and are rapidly activated to initiate outside-in signaling [9–11]. Integrin-dependent signals are required for the formation of the Src-FAK complex at the actin-rich adhesion sites [12]. The downstream effectors of this signaling complex such as cytoskeletal protein paxillin, Rac specific exchange factors Vav1 and Vav3, and the molecular adaptor, SLP-76, regulate platelet spreading [13–15]. However, the contribution of α_IIb_ in the process of outside-in signaling is not well understood.

Our laboratory has identified a calcium- and integrin-binding protein 1 (CIB1) that specifically interacts with the α_IIb_ cytoplasmic tail [16]. Upon platelet activation by various physiological agonists, CIB1 translocates to the cytoskeleton in a time-dependent manner, which parallels the translocation of α_IIb_ to the cytoskeleton [17]. We have also shown that the interaction of CIB1 with α_IIb_ is required for platelet spreading on immobilized Fg [18–20]. Here we show that outside-in signaling-induced calcium rise and filopodia formation precedes CIB1 binding to α_IIb_. Blockade of this interaction inhibits recruitment of FAK and subsequent activation of both FAK and c-Src, which is needed for platelet spreading. Our results show for the first time that α_IIb_ cytoplasmic tail regulates c-Src activity via CIB1 during outside-in signaling.

## Materials and methods

### Antibodies and reagents

The monoclonal antibody anti-CIB1 was generated in our laboratory as described [16]. Purified human Fg was purchased from Enzyme Research Laboratories, Inc (South Bend, IN); BAPTA-AM, DMSO, U73122, an inhibitor of phospholipase C (PLC) and cytochalasin D were from Sigma (St. Louis, MO). Polyclonal antibodies against FAK, c-Src, and α_IIb_ were purchased from Santa Cruz Biotechnology (Santa Cruz, CA). Anti- pY^418^Src and anti-pY^397^FAK was obtained from BioSource, (Carmillo, CA). Additionally, anti-α_IIb_ (SEW8) was a generous gift from Dr. Peter J. Newman (Blood Research Center, Milwaukee, WI). PP2 was obtained from Calbiochem (San Diego, CA). Sequence and synthesis of α_IIb_ cytoplasmic domain peptide and the corresponding scrambled peptide were described [18]. Other chemicals, unless indicated, were of analytical grade purchased from Sigma.

### Ethical statement

Whole blood was drawn by venipuncture from healthy aspirin-free volunteers, older than 18 years, after obtaining written informed consent prior to initiating the study. Approval was obtained from Thomas Jefferson University, Philadelphia; Institutional Review Board for these studies according to the Declaration of Helsinki. Approval for animal experiments was received from the Thomas Jefferson University Institutional Animal Care and Use Committee.

### Human platelet preparation

Approximately six parts of blood was collected in one part of acidified citrate dextrose (pH 4.5) as an anticoagulant. Platelet-rich plasma was obtained from which platelets were isolated and washed as described [21, 22]. Washed platelet suspension was adjusted to 2 X 10^8^ platelets /ml as required.

### Mouse platelet preparation

*Cib1*^-/-^ mice of C57BL/6 genetic background, 10–14-week-old were used in these studies as described previously [23]. Isolation of murine platelets was prepared as described [24].

### Incorporation of peptides and antibodies in the platelets

For the incorporation of peptides and antibodies into the platelets, DMSO method was used as described [18]. Platelets were monitored periodically using a phase-contrast microscope to assess spreading as a readout for platelet functionality after treatment.

### Immunofluorescence microscopy

Immunofluorescence studies were performed following the procedure described [19]. In case of inhibitors treatment, platelets were incubated in the presence or absence of inhibitors and then allowed to spread on immobilized BSA or Fg for 45 min and processed as described [18]. Stained samples were analyzed using a Zeiss LSM 510 laser-scanning microscope (Thornwood, NY). Differential interference contrast microscopy (DIC) images of the platelets on coverslips were captured using Zeiss Axioskop II light microscope using Zeiss Plan-Apochromat 100x/1.4NA oil immersion lens. The images were digitally recorded using Zeiss AxioVision software as described previously [20]. Images were processed using Adobe Photoshop Software.

### Immunoprecipitation and western blotting

Washed platelets (4.0 x 10^8^ platelets/ml) after appropriate treatments as indicated were exposed to 3% BSA or immobilized Fg (100 μg) for ~45 min and then lysed using lysis buffer (1% Nonidet P-40, 150 mM NaCl, 50 mM Tris-HCl pH 7.5, 10 mM sodium orthovanadate, 10 μg/ml leupeptin, 10 μg/ml aprotinin, 1 mM phenylmethanesulfonyl fluoride (PMSF), and 1 mM NaF) for 30 min on ice, and then centrifuged at 13,000 rpm for 10 min at 4°C and the lysate was pre-cleared with isotype-specific antibody. Pre-cleared lysate (500 μg/ml) were immunoprecipitated with overnight incubation at 4°C with appropriate primary antibodies as indicated followed by protein G-Sepharose beads (Amersham Biosciences, Piscataway, NJ). Immunocomplex-captured beads were washed with lysis buffer without inhibitors, and boiled in 2X Laemmli sample buffer and western blotted as described [25]. Band density was quantitated using Bio-Rad Gel-Doc scanning software (Richmond, CA) and Image J Software.

### FAK activation assay

For the murine FAK activation experiments, 60 mm non-tissue culture petri dishes were coated with 2 μg/ml and 100 μg/ml of immobilized Fg or 3% BSA as a control at 37°C for 1 h. *Cib1*^+/+^ and *Cib1*^-/-^ mouse platelets containing 2x10^8^ platelets/ml (500 μl/dish) were exposed to immobilized Fg or BSA for 90 min at 37°C. In a separate set of experiments human platelets (2x10^8^ platelets/ml) were incorporated with DMSO or with antibodies or peptides as indicated and then allowed to spread on 100 μg/ml of immobilized Fg coated dishes for 45 min. The platelets were lysed immediately using ice-cold lysis buffer as described [20]. Platelet lysates were subjected to immunoprecipitation with anti-FAK or isotype–specific control IgG and processed as described in western blotting.

### *In vitro* kinase assay

Pre-cleared lysates from BSA and Fg attached platelet samples (500 μg/ml) were immunoprecipitated as described above using anti-CIB1 or cIgG and the immunoprecipitates were used to perform *in vitro* kinase assay. The immunocomplex beads were washed with kinase assay buffer (20 mM Tris-HCl [pH 7.5], 10 mM MgCl_2_, 2 mM CaCl_2_, and 1 mM DTT). The kinase assay was initiated by adding kinase buffer containing [γ-^32^P] ATP (2 μCi) and myelin basic protein (MBP) as a substrate following previously described procedure [21]. The reactions were stopped by adding 2X Laemmli sample buffer and were separated by SDS-PAGE, transferred to a PVDF membrane and subjected to autoradiography. The same membrane was reprobed with the specific antibodies as indicated to visualize proteins of interest by western blotting. Experiments were repeated more than three times, using platelets from different healthy individuals.

### c-Src kinase assay

Src kinase assay was performed using c-Src Assay Kit following the manufacturer’s protocol (Upstate Biotechnology, Lake Placid, NY). Briefly, immunoprecipitates of anti-CIB1 or cIgG from BSA and Fg-adherent platelet lysates were equilibrated with the kinase buffer containing peptide substrate (KVEKIGEGTYGVVYK). The reaction was initiated by adding 10 μL of [γ-^32^P] ATP and the mixture was incubated for 10 min at 30°C with agitation and the reactions were stopped by adding 20 μL of 40% trichloroacetic acid (TCA) to precipitate peptide. The samples were spotted onto P81 phosphocellulose paper, washed with 0.75% phosphoric acid followed by a final wash with acetone. The individual paper was transferred to a scintillation cocktail vial and the radioactivity was counted using Beckman Scintillation Counter.

### Statistical analysis

Statistical analysis of the data was performed using Sigma plot software. Student’s paired *t* test (mean ± standard error of the mean) were performed to determine statistical significance. *P≤* 0.05 was regarded as statistically significant. Each experiment was repeated independently at least three times.

## Results

### Ca^2+^ dependent interaction of CIB1 with the α_IIb_β_3_ is required for platelet spreading on immobilized Fg

**Fig 1:**
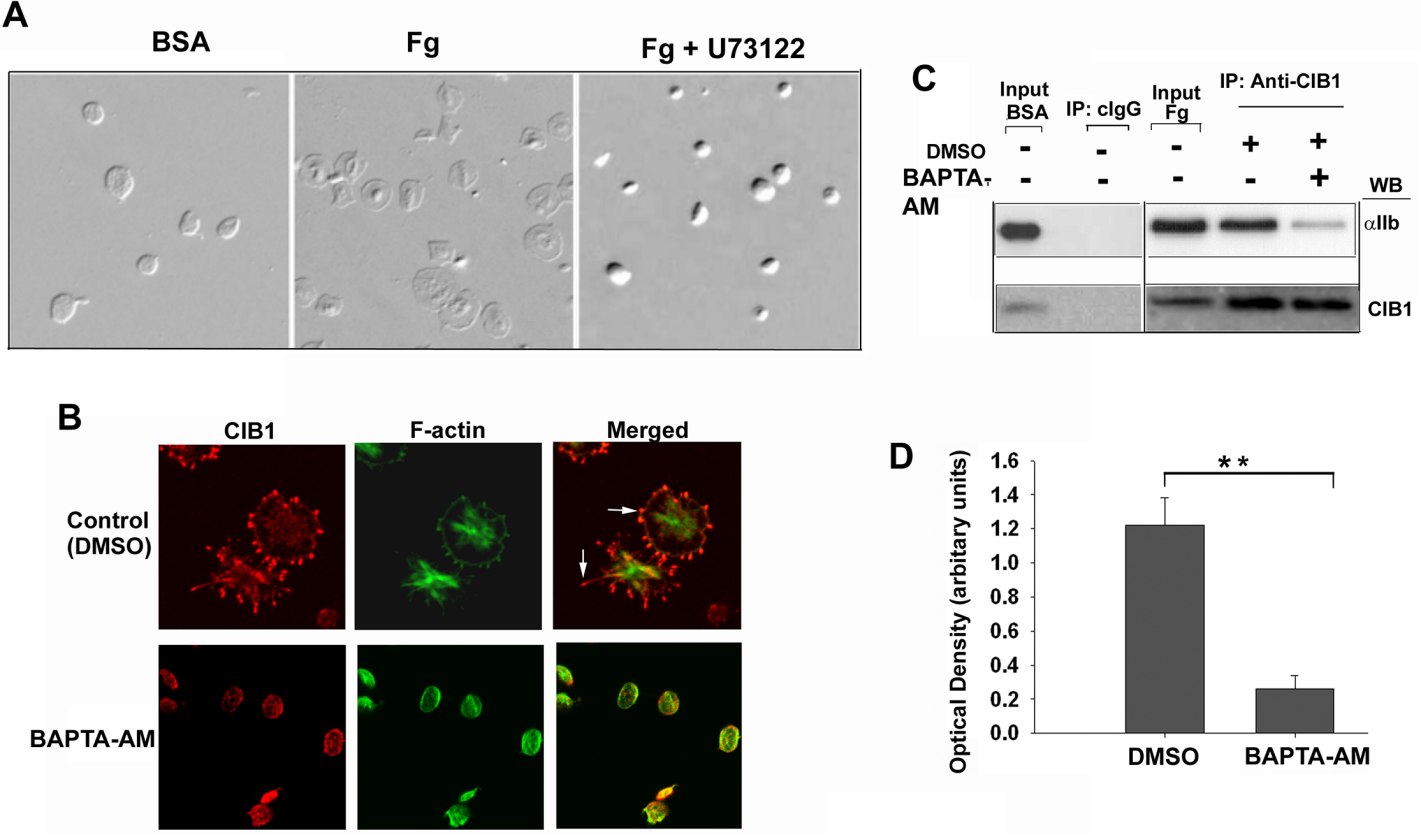
Interaction of CIB1 with α_IIb_β_3_ requires intracellular calcium rise. (A) DIC images of human platelets treated as indicated. (B) Confocal images of platelets in suspension pre-treated with or without BAPTA-AM (50 μM) and then allowed to spread on immobilized Fg-coated coverglass for 45 min. Accumulation of CIB1 at the filopodia and the membrane periphery are indicated by arrows; original magnification, X 1600. (C) Western blot analysis of cIgG or anti-CIB1 immunoprecipitates of lysates of platelets treated as in B. Input represents sample of platelet lysate before immunoprecipitation. α_IIb_ bands are shown in upper blot. Same blot was reprobed with anti-CIB1 to ensure an equal amount of protein in the immunoprecipitates (lower blot). (D) Densitometric analysis of coimmunoprecipitated α_IIb_ bands normalized to corresponding CIB1 band (**P<0.*01*). Error bars indicate mean ± SEM of at least three independent experiments.

When resting platelets are exposed to immobilized Fg, filopodia are formed first followed by lamellipodia through outside-in signaling. We have previously reported that interaction of CIB1, a calcium-binding protein, with α_IIb_ is required for lamellipodia [18]. We therefore evaluated if intracellular Ca^2+^ is required for association of CIB1 with α_IIb_. As expected, vehicle-treated platelets remained discoid when exposed to BSA, but spread well on immobilized Fg. Inhibition of PLC activity, which is required for generation of IP3 leading to intracellular Ca^2+^ release, completely abolished platelet spreading when exposed to Fg, keeping the platelets in a discoid shape (Fig 1A). Platelet adhesion to Fg was not affected. We next determined if filopodia formation and CIB1 localization at the filopodia requires intracellular Ca^2+^ rise. Immunofluorescence staining indicated that platelets pre-treated with DMSO alone spread well on Fg and CIB1 was accumulated at the filopodia (Fig 1B). However, platelets pre-treated with BAPTA-AM, a Ca^2+^ chelator, remained discoid without any filopodia formation and CIB1 staining was diffuse in the cytoplasm (Fig 1B). A co-immunoprecipitation experiment indicated that CIB1 interaction with α_IIb_ is greatly reduced in the presence of BAPTA-AM (Fig 1C). Quantitation of this data showed that a six-fold reduction was observed in the association of CIB1 with α_IIb_ in the presence of BAPTA-AM (Fig 1D). These results suggest that Fg binding to α_IIb_β_3_ initiates an intracellular Ca^2+^ rise, which is required for filopodia formation as well as CIB1 binding to α_IIb_ during outside-in signaling.

### Filopodia formation precedes CIB1 association with α_IIb_ and accumulation at the filopodia

**Fig 2:**
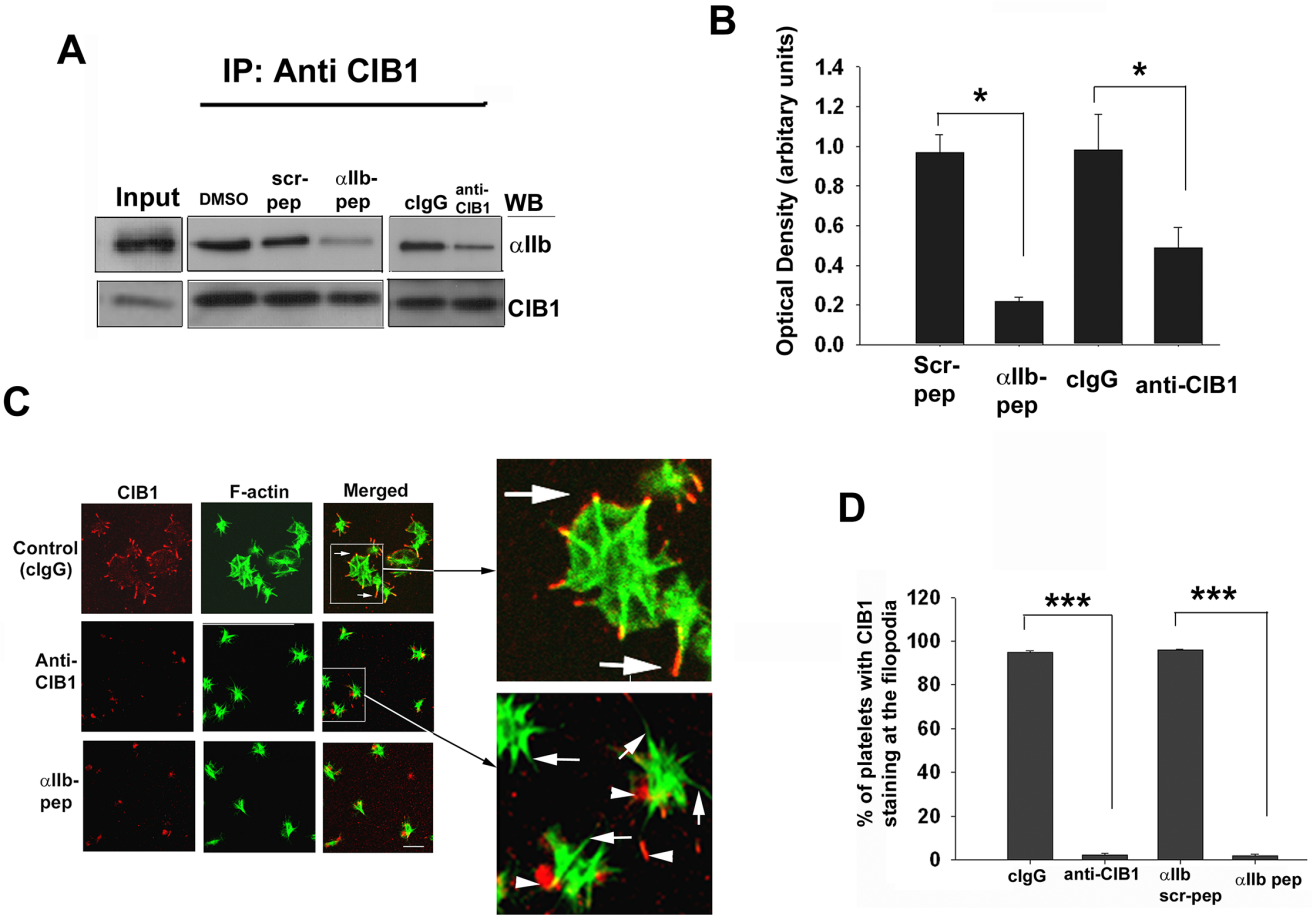
Association of CIB1 with α_IIb_ is needed for platelet spreading but not filopodia formation. (A) Western blot analysis of anti-CIB1 immunoprecipitates of lysates from platelets incorporated with either α_IIb_ peptide (2.5 μM) or anti-CIB1 antibody (0.1 mg/ml) as indicated. DMSO, scrambled α_IIb_ peptide (2.5 μM) and irrelevant isotype specific antibody (cIgG, 0.1 mg/ml) were used as control. Upper blot shows the co-immunoprecipitation of α_IIb_. Same blot was reprobed with anti-CIB1 to ensure equal amount of protein in the immunoprecipitates (lower blot). Input represents sample of platelet lysate prior to immunoprecipitation. (B) Quantitation of α_IIb_ bands from (A) normalized to corresponding CIB1 band. (*P<0.*05)* (C) Confocal images of platelets incorporated with α_IIb_ peptide, anti-CIB1 or corresponding controls DMSO, scrambled α_IIb_ peptide, or cIgG and were allowed to spread on Fg for 45 min and stained for F-actin; boxed area is enlarged to visualize colocalization (arrows); non-specific red speckles are shown by arrowheads; original magnification, X 1600. (D) Quantitation of platelets from C showing CIB1 staining at the filopodia (***P<0.*001*). Error bars indicate mean ± SEM of at least three independent experiments.

To determine if Ca^2+^-dependent CIB1 binding to α_IIb_ is necessary for filopodia formation, we used a previously described method to introduce exogenous proteins/peptides into the resting platelets [18]. Although it is known that incorporation of palmitoylated peptide corresponding to α_IIb_ cytoplasmic tail activates platelets [26], we found that unmodified peptide corresponding to α_IIb_ tail did not activate platelets. To eliminate the possibility that the α_IIb_ peptide may affect other α_IIb_ domain binding proteins, we also incorporated a CIB1 function-blocking antibody. We first determined the effect of incorporation of the unmodified α_IIb_ peptide or anti-CIB1 on the interaction of CIB1 with α_IIb_ during outside-in signaling. Immunoprecipitation experiments performed showed that a substantial amount of association of CIB1 with α_IIb_ occurred in mock-treated platelets (Fig 2A). This association was significantly inhibited (>80%) by the incorporation of the α_IIb_ peptide, but not its scrambled peptide, which was used as a control (Fig 2A & 2B). This is further supported by the finding that interaction of CIB1 with α_IIb_ is significantly reduced (>50%) in the presence of CIB1 function-blocking antibody, compared to the presence of a control isotype specific antibody (cIgG) (Fig 2A & 2B). The greater extent of reduction in the association of CIB1 with α_IIb_ in the presence of the α_IIb_ peptide could be due to the higher efficiency of incorporation of a molar amount of peptide compared to the antibody. We next evaluated the effect of α_IIb_ peptide or antibody on CIB1 localization during platelet spreading on immobilized Fg. Platelets mock-treated or treated with cIgG or scrambled peptide progressed to the characteristic fully spread morphology with CIB1 staining along the membrane (Fig 2C). Consistent with our previous finding [18], the incorporation of α_IIb_ peptide or anti-CIB1 into the platelets had no effect on platelets attaining a spiky morphology. However, we found that CIB1 staining was barely detectable at the filopodia (Fig 2C). Interestingly, platelet adhesion to Fg was not inhibited by the incorporation of either anti-CIB1 or α_IIb_ peptide as determined by the number of adherent platelets per field (Fig 2C). Upon quantification of the spiky platelets showing presence of CIB1 at the filopodia, we found that the incorporation of anti-CIB1 or α_IIb_ peptide completely abolished the CIB1 accumulation at the filopodia as compared to control platelets (Fig 2D). These results indicate that filopodia formation occurs prior to the interaction between CIB1 and α_IIb_.

### Dynamic cytoskeletal rearrangement is required for CIB1–dependent activation of FAK

**Fig 3:**
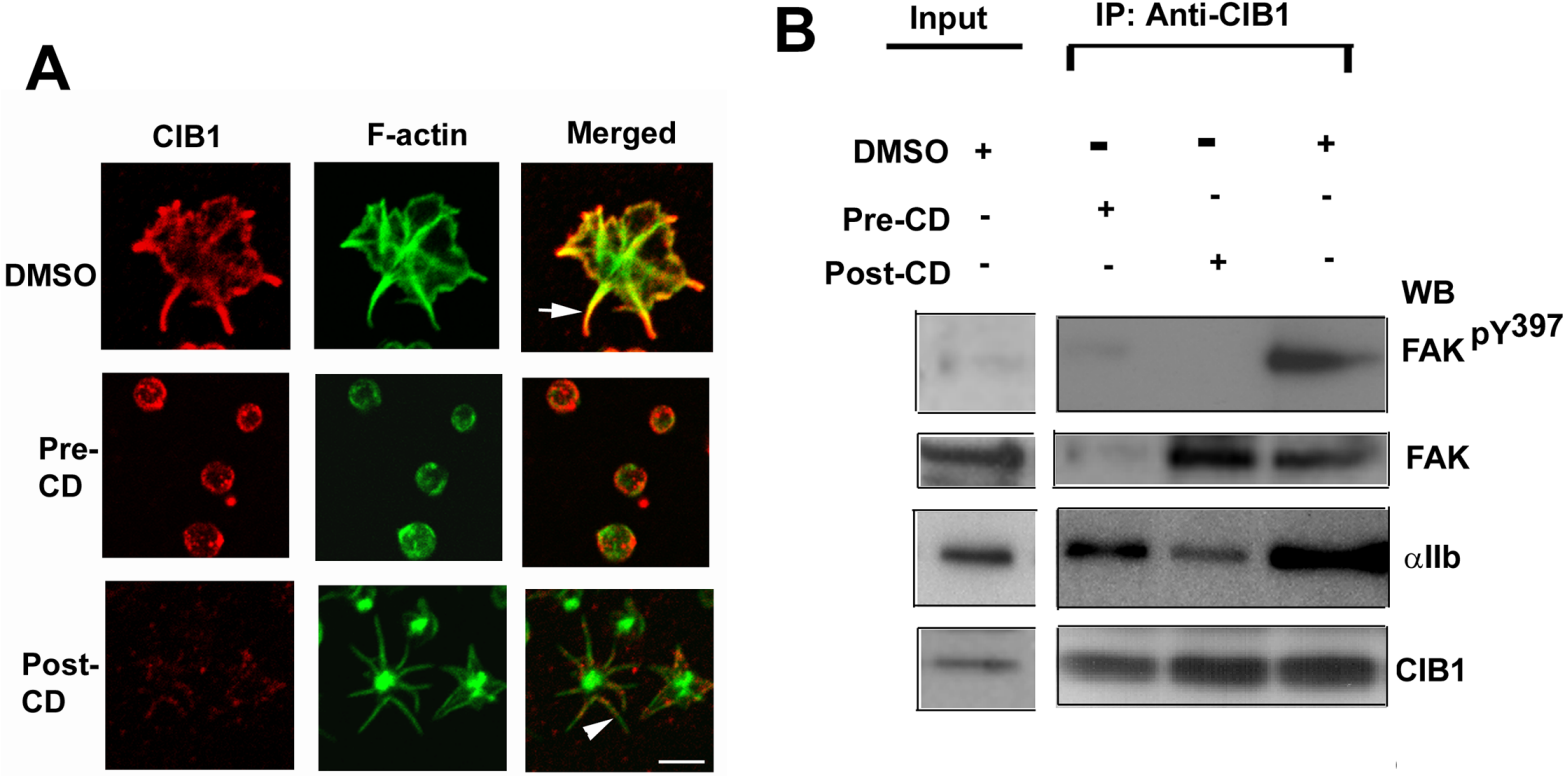
Dynamic cytoskeletal rearrangement is required for activation of FAK. (A) Confocal images of vehicle treated (DMSO) platelets, of platelets pre-treated with cytochalasin D (10 μM) for 15 min (Pre-CD) and then allowed to spread on Fg for 30 min, of platelets allowed to spread for 30 min prior to treatment with cytochalasin D for additional 15 min (Post-CD). Arrow indicates CIB1 staining at the tip of the filopodia. Arrowhead indicates filopodia lacking CIB1 staining; original magnification, X 1600. (B) Western blot analysis of anti-CIB1 immunoprecipitates of lysates from platelets treated as in A. Top blot shows phosphorylated Y^397^FAK. The blot was reprobed for total FAK co-immunoprecipitated with CIB1 (upper middle), and for CIB1 to ensure equal amount of protein in the immunoprecipitates (bottom). Samples from B were also blotted for α_IIb_ (lower middle). Shown are the representative blots from three separate experiments performed independently.

We observed that spatial distribution of CIB1 changes as platelet spread on immobilized Fg. We therefore sought to determine the role of actin cytoskeleton on CIB1 localization. Treatment of platelet with DMSO as vehicle control did not affect platelet spreading or localization of CIB1 (Fig 3A). When platelets were pretreated with cytochalasin D, an inhibitor of actin polymerization, platelets remained discoid, failing to obtain a spiky or spread morphology as expected, and CIB1 showed a typical submembranous localization (Fig 3A). We next asked if dynamic cytoskeletal rearrangement is necessary for CIB1 localization at the filopodia. Cytochalasin D treatment of platelets after they attain filopodial morphology (15 min exposure to immobilized Fg) caused most of the F-actin staining to accumulate at the center of the platelets, they maintained a spiky morphology with abundant filopodia. However, CIB1 accumulation was substantially reduced at the filopodia (Fig 3A) clearly suggesting that dynamic cytoskeletal rearrangement is necessary for the CIB1 localization to the filopodia.

CIB1 is known to co-immunoprecipitate with FAK in a platelet activation-dependent manner. [19] This association appears to be indirect since we were not able to demonstrate physical association of FAK with CIB1 in a purified system. Since dynamic cytoskeletal rearrangement is necessary for FAK activation [12], we investigated the effect of cytochalasin D treatment pre- and post-adhesion of platelets to Fg on association of CIB1 with FAK. When assessed if the association of CIB1 with α_IIb_ had occurred in these conditions, we found that a significant amount of α_IIb_ was associated with CIB1 in vehicle-treated (DMSO) as well as treated with cytochalasin D pre- and post-adhesion. In DMSO-treated platelets, CIB1 was found to associate with active FAK (phosphorylated on Y^397^ residue) (Fig 3B). In platelets pretreated with cytochalasin D, CIB1 failed to associate with FAK (Fig 3B) suggesting that initial cytoskeletal rearrangement is necessary for their interaction. However, post-adhesion treatment showed CIB1 association with inactive FAK as indicated by the absence of tyrosine phosphorylation on Y^397^ residue (Fig 3B). These results suggest that during outside-in signaling, association of CIB1 with the α_IIb_β_3_ complex occurs prior to the recruitment of FAK and its activation, which requires dynamic cytoskeletal rearrangement.

### Association of CIB1 with α_IIb_ is prerequisite for the recruitment and activation of FAK

**Fig 4:**
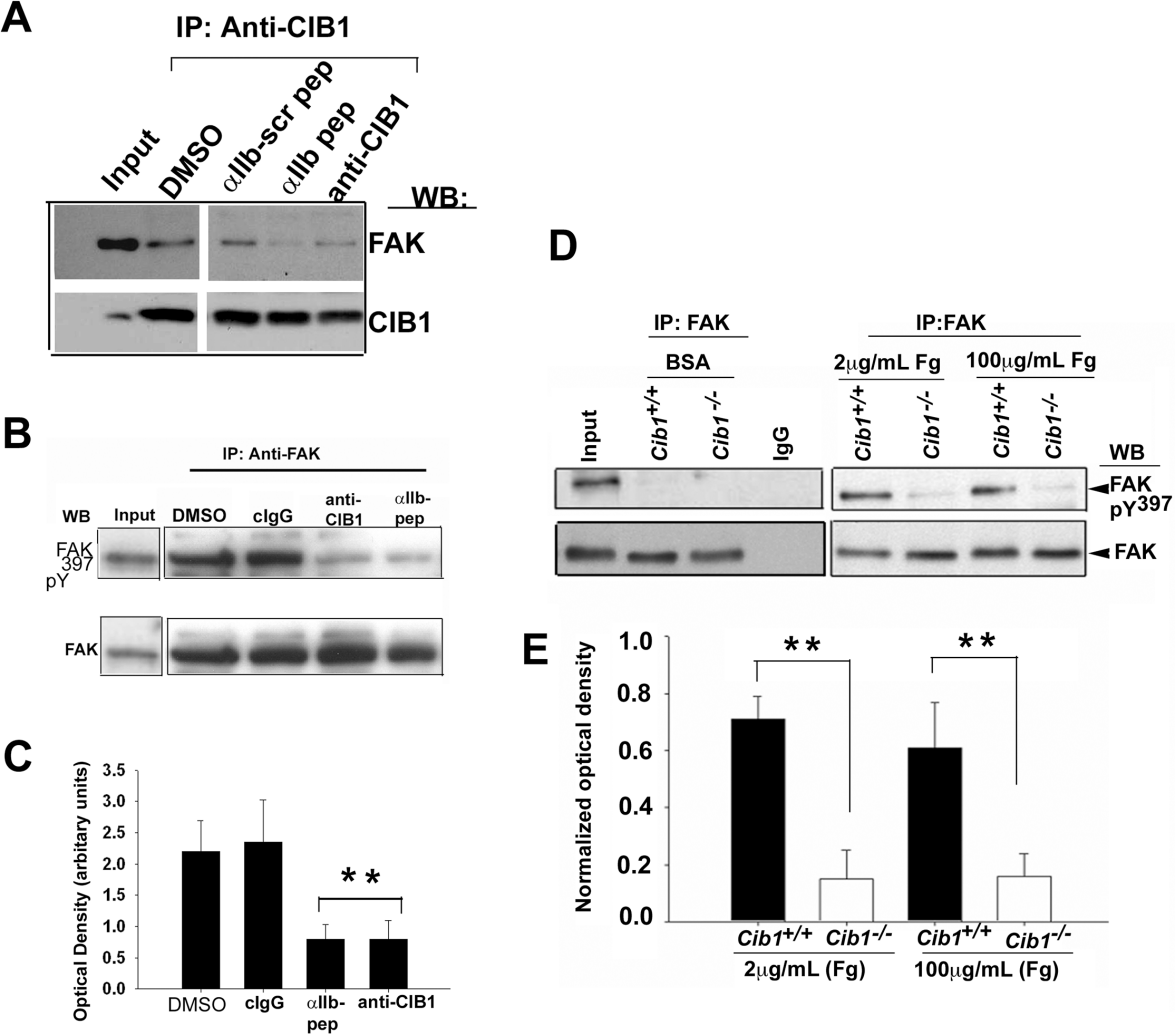
Association of CIB1 with α_IIb_ is essential for the recruitment and activation of FAK. (A) Western blot analysis of anti-CIB1 immunoprecipitates of lysates of platelets incorporated with anti-CIB1 or α_IIb_ peptide and corresponding scrambled peptide or DMSO and allowed to spread on Fg. Input represents sample of platelet lysates prior to immunoprecipitation. FAK bands are shown in upper blot. Same blot was reprobed with anti-CIB1 to ensure an equal amount of protein in the precipitates (lower blot). (B) Western blot analysis of anti-FAK immunoprecipitates of lysates of platelets treated as above. cIgG was used as control. Activated FAK bands identified using anti-pY^397^ are shown in upper blot. The same blot was reprobed for total FAK to ensure an equal amount of protein in the precipitates. (C) Quantitation of optical density of phosphorylated FAK bands from B, normalized to corresponding total FAK band (**P<0.*01*). Error bars indicate mean ± SEM of at least three independent experiments. (D) Western blot analysis of anti-FAK immunoprecipitate of lysates of *Cib1*^*+/+*^ *and Cib1*^*-/-*^ mouse platelets exposed to BSA or immobilized Fg. Activated FAK bands identified using anti-pY^397^ are shown in upper blot. The same blot was reprobed for total FAK to ensure equal protein in the precipitates. (E) Quantitation of optical density of phosphorylated FAK bands from D, normalized to corresponding total FAK band. (**P<0.*01*).

We next asked if association of FAK with CIB1 is disrupted by α_IIb_ peptide or anti-CIB1 in platelets. Consistent with our previous finding, FAK readily and specifically co-immunoprecipitated with CIB1 from lysates of platelets exposed to Fg in the presence of DMSO [19]. Incorporation of α_IIb_ peptide, but not scrambled peptide significantly blocked co-immunoprecipitation of FAK (Fig 4A). Incorporation of anti-CIB1 also blocked co-immunoprecipitation of FAK with CIB1 (Fig 4A). These results suggest that association of CIB1 and α_IIb_ is necessary for the recruitment of FAK.

Because disruption of CIB1 and α_IIb_ interaction blocked platelet spreading, we asked whether this effect is through inhibition of FAK activation. The incorporation of anti-CIB1 or α_IIb_ peptide substantially blocked FAK activation (Fig 4B), whereas incorporation of either the cIgG or control treatment (DMSO) had no effect on FAK activation induced by platelet adhesion to Fg (Fig 4B). Densitometric analysis indicated that ~70% of FAK activation is inhibited by incorporation of either α_IIb_ peptide or anti-CIB1 compared to cIgG or DMSO (Fig 4C). These results suggest that interaction between CIB1 and α_IIb_ is needed for FAK activation during platelet spreading on immobilized Fg. This was further demonstrated by the use of *Cib1*^*-/-*^ platelets. FAK was immunoprecipitated from the lysates of WT and *Cib1*^*-/-*^ platelets exposed to immobilized BSA or Fg for 1 h and western blotted for phospho-Y^397^FAK. As expected, abundant FAK was immunoprecipitated from lysates of platelet exposed to BSA; it was not phosphorylated. On the other hand, FAK that was immunoprecipitated from WT platelets exposed to both 2 μg/ml and 100 μg/ml of Fg was phosphorylated. Interestingly, FAK immunoprecipitated from lysates of *Cib1*^*-/-*^ platelets exposed to Fg lacked phosphorylation (Fig 4D & 4E). These results indicate that during outside-in signaling association of CIB1 with α_IIb_ is a prerequisite for FAK activation.

### CIB1 association with α_IIb_ and subsequent FAK activation is required for the activation of c-Src

**Fig 5:**
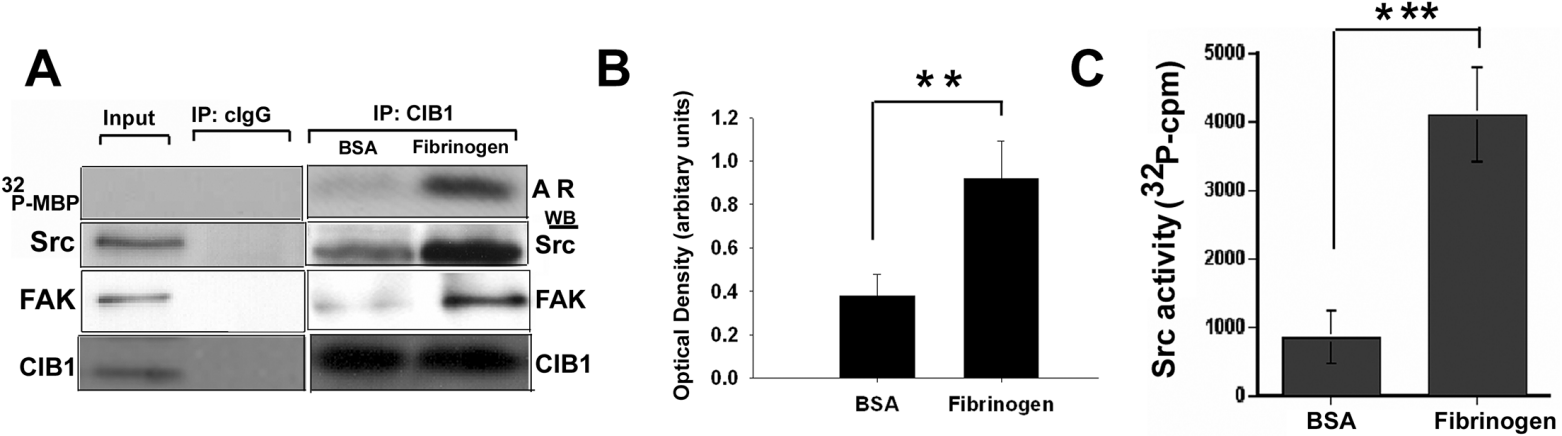
Association of c-Src with CIB1-FAK complex augments its activity. (A) Washed platelets were exposed to BSA or adhered to Fg for 45 min. The pre-cleared detergent lysates of these platelets were immunoprecipitated with anti-CIB1 or cIgG. Autoradiogram (AR) of MBP phosphorylation (upper panel). Western blot of immunoprecipitates probed with anti-Src (upper middle), and with anti-FAK (lower middle). The membrane was reprobed with anti-CIB1 to ensure equal amount of protein in the precipitates (lower panel). (B) Densitometric analysis of co-immunoprecipitated c-Src (upper middle blot in (A) (**P < 0.*01*). Error bars indicate mean ± SEM of at least three independent experiments. (C) Src kinase activity in CIB1 immunoprecipitates from platelets attached to Fg compared to BSA (***P<0.*001*).

It is well documented that c-Src is constitutively associated with β_3_ and is activated by FAK as a result of binding to phosphorylated Y^397^. Since our results indicate that CIB1 binding to α_IIb_ increases Y^397^ phosphorylation of FAK, we asked if c-Src is also activated in the CIB1 complex. An immunocomplex kinase assay performed with MBP as a substrate indicated that CIB1 immunoprecipitate from platelets attached to Fg contains a high level of MBP phosphorylating activity as compared to immunoprecipitates from platelets attached to BSA (Fig 5A). Control immunoprecipitate (cIgG) did not show any kinase activity. We next sought to determine if c-Src was present in the CIB1 immunoprecipitates. We found that platelets attached to Fg had over two-fold more c-Src associated with CIB1 compared to platelets on BSA (Fig 5B). This increased level of c-Src is most likely due to the increased association of α_IIb_β_3_ with CIB1. As expected from our previous findings, we also found that an increased amount of FAK was associated with CIB1 on Fg (Fig 5A) [19]. Furthermore, we found that the amount of c-Src immunoprecipitated with CIB1 from lysates of platelets attached to Fg was ~two-fold (Fig 5B), the c-Src activity was increased almost five-fold (Fig 5C) as compared to control platelets exposed to BSA. These results suggest that CIB1 recruits and modulates FAK phosphorylation on Y^397^, which allows the activation of c-Src during α_IIb_β_3_ outside-in signaling.

### CIB1 localization to the filopodia is independent of but required for c-Src kinase activity

**Fig 6:**
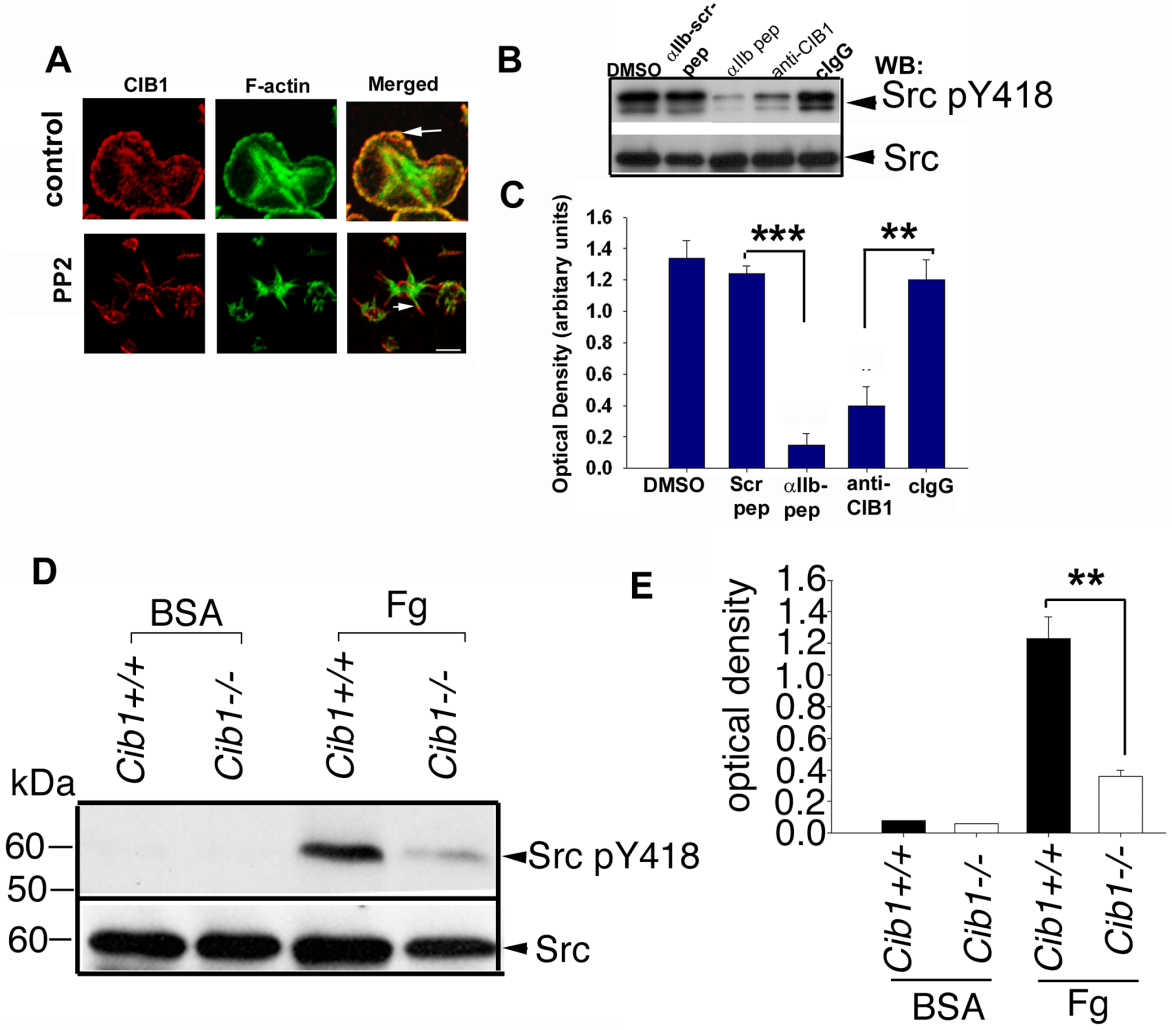
CIB1 association with α_IIb_ is required for c-Src kinase activity. (A) Confocal images of washed platelets pretreated in suspension with DMSO or Src kinase inhibitor PP2 (10 μM) for 20 min and allowed to spread on Fg for 45 min. Arrows indicate the localization of CIB1. (B) Western blot analysis of lysates from platelets incorporated with either α_IIb_ peptide or anti-CIB1 antibody; DMSO, scrambled α_IIb_ peptide, and cIgG as control probed with anti-Src pY^418^. The same blot was reprobed with anti-Src antibody to show the equal protein loading. (C) Densitometric analysis of (B). ***P< 0.*001* and **P<0.*01*. (D) Western blot analysis of lysates prepared from *Cib1*^*+/+*^ and *Cib1*^*-/-*^ mouse platelets. Activated c-Src bands identified using anti-c-Src-pY^418^ are shown in upper blot. The same blot was reprobed for total c-Src to ensure equal amount of protein loaded. (E) Densitometric analysis of c-Src-pY^418^ bands from D; normalized to corresponding total c-Src band (**P<0.*01*) from three independent experiments.

Since a population of c-Src co-immunoprecipitates with CIB1 during outside-in signaling, we asked if c-Src activity is responsible for the specific localization of CIB1 at the filopodia. To test this, we pretreated platelets with known Src-family kinase inhibitor PP2 and allowed them to spread on immobilized Fg. Consistent with the previously published reports, treatment with PP2 abolished platelet spreading, but did not affect filopodia formation. CIB1 accumulation to the filopodia was also not affected by PP2 (Fig 6A), suggesting that Src kinase activity is not required for filopodia formation and CIB1 localization to the filopodia. The observed spiky phenotype was similar to that observed when interaction of CIB1 with α_IIb_ was inhibited (Figs 2C & 6A). We therefore asked if the interaction of CIB1 with the α_IIb_ is required for c-Src activation. We found that incorporation of either α_IIb_ peptide or anti-CIB1 dramatically inhibited c-Src activation as assessed by phosphorylation of Y^418^ residue, whereas the controls had no effect (Fig 6B). Quantitation of this data revealed that both α_IIb_ peptide as well as anti-CIB1 significantly inhibited c-Src activation during outside-in signaling; with the α_IIb_ peptide being more potent, inhibiting more than eight-fold, whereas the anti-CIB1 inhibited about three-fold possibly due to the lower efficiency of incorporation (Fig 6C). To further evaluate the need of CIB1 for c-Src activation during outside-in signaling, we assessed the activation of c-Src in *Cib1*^*+/+*^ and *Cib1*^*-/-*^ platelets. Platelets exposed to BSA showed no activation of c-Src (Fig 6D). As expected *Cib1*^*+/+*^ platelets showed robust activation of c-Src when exposed to Fg. However, in *Cib1*^*-/-*^ platelets c-Src activation was significantly attenuated upon exposure to Fg (Fig 6D & 6E). Taken together, these results clearly indicate that c-Src activity is not required for filopodia formation and CIB1 to localize to the filopodia and also strongly suggest that interaction of CIB1 with α_IIb_ and recruitment of FAK is necessary for the activation of c-Src kinase during outside-in signaling.

## Discussion

Binding to Fg, platelet α_IIb_β_3_ initiates a cascade of events that are essential for platelet spreading and clot retraction [1–6]. Although extensive advances have been made in understanding the signaling process regulated by α_IIb_β_3_, much remains to be understood. Our systematic study reported here provides, for the first time ever, detailed mechanistic insight into the early signaling events that occur upon ligand binding to the α_IIb_β_3_, without interference from other signaling pathways.

**Fig 7:**
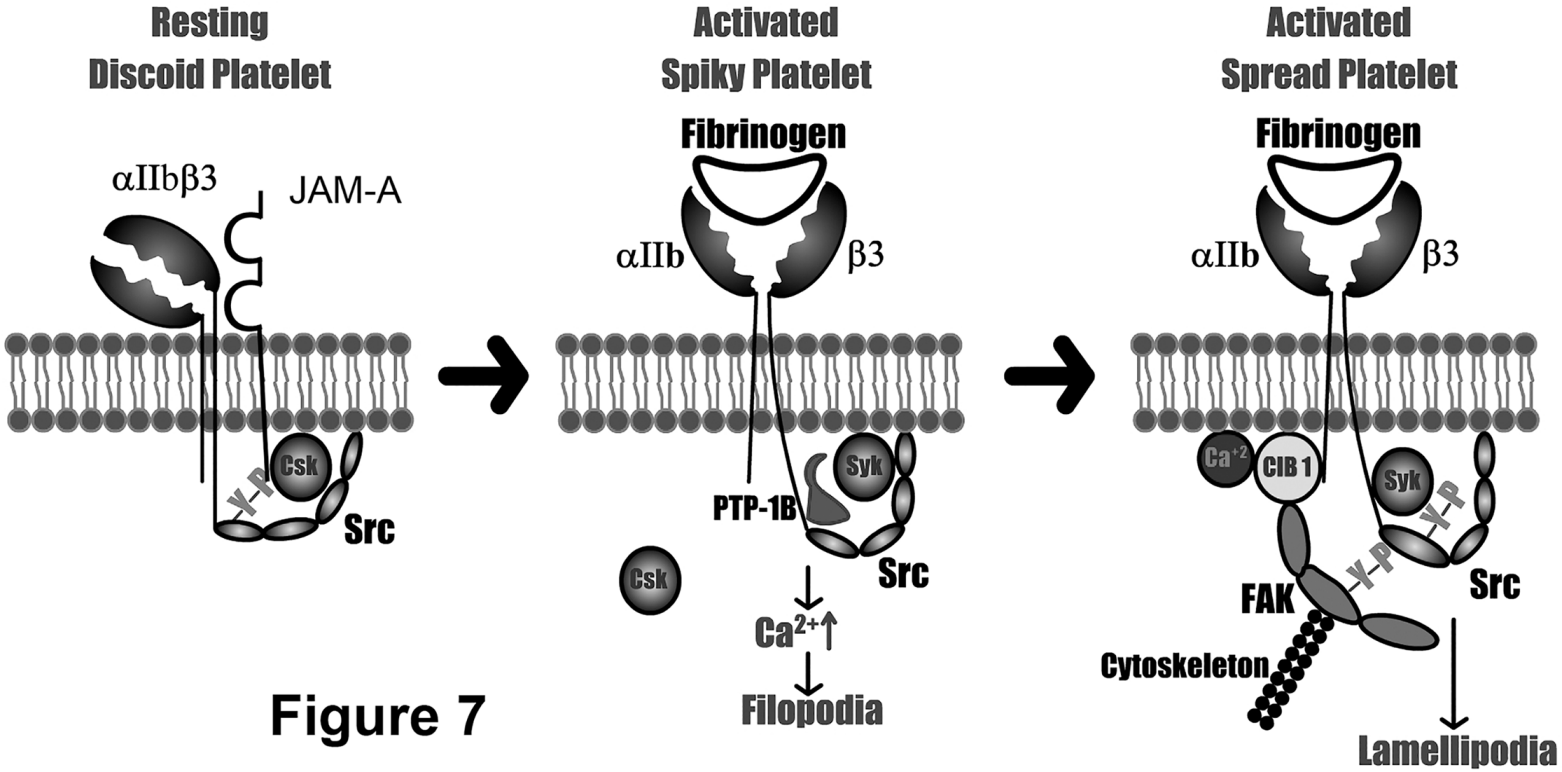
Schematic representation of the initial steps of outside-in signaling through α_IIb_β_3_. Inactive α_IIb_β_3_ in resting platelets complexed with JAM-A (left). Initial steps of outside-in signaling initiated by Fg binding to α_IIb_β_3_ prior to the association of CIB1 leading to filopodia formation (middle). Signaling complex recruited by CIB1 to the α_IIb_ tail leading to Src activation and platelet spreading (right).

In resting platelets, α_IIb_β_3_ is constitutively associated with c-Src and CSK is recruited to this complex by JAM-A [25, 27]. Fg binding to the activated α_IIb_β_3_ results in dissociation of JAM-A/Csk, followed by dephosphorylation of c-Src by PTP-1B and recruitment of Syk to this complex (Fig 7) [28]. We found that an intracellular calcium rise and filopodia formation through cytoskeletal rearrangement, which contributes to the spiky morphology, occurs very early in this process. Calcium binding to CIB1 results in a conformational change that enables it to associate with α_IIb_ and recruit FAK to this complex (Fig 7). The dynamic cytoskeletal rearrangement which follows is necessary for the activation of FAK. Binding of c-Src to phospho-Y^397^FAK enhances its activation, inducing downstream signaling needed for lamellipodia formation and hence platelet spreading (Fig 7). Our results provide strong evidence for a significant involvement of α_IIb_ tail in the outside-in signaling. This is consistent with previous reports proposing involvement of α_IIb_ tail in outside-in signaling which received very little attention [29, 30].

Since the discovery of CIB1 as α_IIb_ binding protein, it has been reported to have a variety of functions, starting from the megakaryocytes to the aggregated platelets [18, 19, 31–33]. We have previously shown that CIB1 is a key regulator of α_IIb_β_3_ outside-in signaling [18, 19]. It is also possible that CIB1 may participate in regulating platelet activation [32]. To avoid the interference from agonist-induced inside-out signaling, we used aspirin-treated platelets in the presence of apyrase to study signaling pathways induced when α_IIb_β_3_ binds to immobilized Fg. This system has been widely used to delineate the outside-in signaling mechanisms [34, 35]. Our results indicate that a rise in intracellular Ca^2+^ and rearrangement of the actin cytoskeleton to form filopodia occurs prior to the interaction of CIB1 with α_IIb_. Blockade of CIB1 binding to the α_IIb_ does not affect filopodia formation, but abolishes localization of CIB1 at the filopodia in platelets adherent to immobilized Fg. This finding is supported by the observations that CIB1 preferentially binds activated α_IIb_β_3_ as observed in an *in vitro* binding assay [36] and that CIB1 translocates to the cytoskeleton along with α_IIb_β_3_ in an aggregation-dependent manner, which could be inhibited by RGDS peptide [17]. Since CIB1 only interacts with α_IIb_ tail of the activated-ligand bound α_IIb_β_3_, we do not expect any interference of CIB1 with binding of talin and/or kindlin.

In addition to binding to the α_IIb_ cytoplasmic domain, CIB1 appears to associate with FAK during outside-in signaling. We have previously shown that CIB1 associates with FAK and together they colocalize to the focal adhesions and tip of the filopodia [19]. Our results suggest that CIB1 binding to α_IIb_β_3_ and activation of FAK is required for c-Src activation. However, it has also been shown that FAK, but not c-Src activation requires dynamic cytoskeletal rearrangement [34]. It is therefore possible that c-Src is partially activated independent of FAK activation, but its full activation requires FAK. This is consistent with the observation that c-Src activity is enhanced upon its binding to the phosphorylated Y^397^FAK [12]. Thus, it is possible that CIB1 recruits FAK to the α_IIb_β_3_ where partially activated c-Src binds to the activated FAK and gets fully activated. This notion is consistent with our previous finding that β_3_ tyrosine phosphorylation is attenuated in *Cib1*^*-/-*^ platelets when spread on Fg, suggesting that CIB1 influences β_3_ phosphorylation-dependent signaling in platelets [20]. Activated c-Src triggers phosphorylation of downstream components such as paxillin, SLP76, Vav1, and Vav3 that initiate lamellipodia formation and platelet spreading [34].

CIB1 is shown to interact with a number of different proteins. However, in addition to α_IIb_ and FAK, only WASP, PAK1, SK1, ASK1, and InsP(3)R are reported to be expressed in platelets [32, 37–40]. WASP binds at the N-terminus of CIB1 as opposed to α_IIb,_ which binds to the C-terminus and is involved in inside-out signaling [33]. CIB1-dependent activation of PAK1 has been shown to inhibit cell spreading and migration [38]. Furthermore, CIB1 inhibits InsP(3)R-induced Ca^2+^ release as well as ASK1 activation [39, 40]. It is therefore unlikely that the observed defect in platelet spreading by incorporation of α_IIb_ peptide or anti-CIB1 is due to blockade of CIB1 interaction with any of these proteins. However, more experiments needed to be performed to definitively rule out these possibilities.

In conclusion, this study clearly defines a role for CIB1 in assembling and activating some important tyrosine kinases that have long been implicated in regulating outside-in signaling. This is consistent with the reported *in vivo* data in which *Cib1*^*-/-*^ mice show defective outside-in signaling and are protected from thrombosis [20]. Our results also shed light on separating early events of outside-in signaling such as Ca^2+^ rise and filopodia formation from FAK and c-Src activation leading to lamellipodia formation. Furthermore, our results provide strong evidence for the involvement of integrin α_IIb_ cytoplasmic tail in the early stages of outside-in signaling.
